# Pregabalin vs. gabapentin in the treatment of neuropathic pain: a comprehensive systematic review and meta-analysis of effectiveness and safety

**DOI:** 10.3389/fpain.2024.1513597

**Published:** 2025-01-07

**Authors:** Víctor Mayoral, Rafael Galvez, Marta Ferrándiz, Xoán Miguéns Vázquez, Carlos Cordero-García, Antonio Alcántara Montero, Concepción Pérez, María Pérez-Páramo

**Affiliations:** ^1^ Pain Unit, Anaesthesiology Department, Hospital Universitari de Bellvitge, Barcelona, Spain; ^2^ Pain Unit, Deparment of Anesthesia, Hospital Virgen de las Nieves, Granada, Spain; ^3^ Pain Unit, Hospital Universitari de La Santa Creu I Sant Pau, Barcelona, Spain; ^4^ Department of Physical Medicine and Rehabilitation, Área Sanitaria de Ourense, Verin e O Barco de Valdeorras, Ourense, Spain; ^5^ Department of Physical Medicine and Rehabilitation, Juan Ramón Jiménez University Hospital, Huelva, Spain; ^6^ Centro de Salud Trujillo, Cáceres, Spain; ^7^ Pain Unit, Hospital de la Princesa, Madrid, Spain; ^8^ Medical Department, Viatris, Madrid, Spain

**Keywords:** pregabalin, gabapentin, neuropathic pain, pain, meta-analysis, systematic review

## Abstract

**Introduction:**

Neuropathic pain is a prevalent and burdensome condition, and both pregabalin and gabapentin are widely used for its treatment. However, there is a lack of clarity regarding their comparative efficacy and safety. This meta-analysis aims to evaluate and compare the effectiveness and safety of pregabalin vs. gabapentin in managing neuropathic pain.

**Methods:**

This study followed PRISMA guidelines and employed the PICOS search strategy. Comparative studies (clinical trials and cohort studies) were included, with patients with neuropathic pain treated either with pregabalin or gabapentin. Primary outcomes assessed were efficacy and safety. Data were extracted from PubMed, Embase, Scopus, and the Cochrane Collaboration Library databases. The risk of bias was evaluated using the Cochrane Review Manager tool. Statistical analysis was performed using Review Manager 5.4.1 software, calculating effect sizes and conducting sensitivity analysis based on medication dosage.

**Results:**

A total of 14 studies with 3,346 patients were analyzed. Pregabalin showed superior results compared to gabapentin in the Visual Analog Scale (VAS) at various time intervals up to 12–14 weeks (SMD −0.47, 95% CI −0.74 to −0.19). The pregabalin group also had significant improvements in SF-12/SF-36/EQ-5D scores (SMD 0.39, 95% CI 0.11–0.68) and experienced more days with no/mild pain (MD 9.00, 95% CI 8.93–9.07) and fewer days with severe pain (MD −3.00, 95% CI −4.96 to −1.04). Pregabalin resulted in lower opioid consumption (OR 0.50, 95% CI 0.33–0.76). Gabapentin had a higher incidence of nausea and vomiting. Sensitivity analysis supported the efficacy of pregabalin.

**Conclusion:**

In conclusion, pregabalin demonstrated superior and faster efficacy in alleviating neuropathic pain than gabapentin did. Additionally, it improved patient-reported outcomes, resulted in lower opioid consumption, and led to fewer adverse events.

**Systematic Review Registration:**

https://www.crd.york.ac.uk/prospero/display_record.php?RecordID=565208, PROSPERO (CRD42024565208).

## 1 Introduction

The prevalence of neuropathic pain in the general population is estimated to range from 3.2% to 10.3%, with an even higher prevalence in cases of diabetic neuropathy, ranging from 23% to 46.5% (1). This type of pain poses a challenge in its treatment and has a significant impact on health-related quality of life, as well as a considerable social burden (2). Additionally, according to a revised definition, neuropathic pain is characterized by directly involving the somatosensory system as a result of a specific disease or lesion, which refines the diagnostic criteria and links these to generally accepted neurological principles, providing a more precise basis for its diagnosis and treatment (3).

Neuropathic pain can be highly disabling, generating limitations in functionality and the well-being of patients. Additionally, there has been significant variation in healthcare costs associated with painful diabetic neuropathy in different databases and age groups, reaching up to $8,500 by year (4). There is a clear need to improve pain control and quality of life in patients suffering from various pathologies where neuropathic pain is present, such as in patients undergoing hemodialysis, where more than 50% of them experience inadequately treated pain (5).

The treatment of these conditions is often palliative, as some of them lack a definitive cure. In this context, numerous drugs have been developed for the management of neuropathic pain, including duloxetine, amitriptyline, gabapentin, and pregabalin, among others. Gabapentin and pregabalin are analogs of gamma-aminobutyric acid (GABA) and share a similar mechanism of action, although they differ in some aspects. Both drugs bind to the α2δ subunit of calcium channels in neurons, but pregabalin exhibits greater affinity and potency in its binding (5, 6). In addition to inhibiting the release of excitatory neurotransmitters, such as glutamate, pregabalin also modulates the release of inhibitory neurotransmitters like GABA, increasing its availability in the central nervous system (6, 7). Although pregabalin and gabapentin share similarities in their mechanisms of action, they exhibit some pharmacokinetic and pharmacodynamic differences. In terms of pharmacokinetics, pregabalin demonstrates higher oral bioavailability, faster absorption, and a more predictable dose-response relationship than gabapentin (6, 7). Pregabalin also undergoes minimal metabolism and is primarily excreted unchanged in the urine, while gabapentin undergoes significant renal elimination and requires dosage adjustments in patients with impaired renal function (6, 7).

Several studies have evaluated the efficacy of gabapentin and pregabalin in the treatment of neuropathic pain, yielding contradictory results. On one hand, it has been observed that gabapentin is more effective, especially at higher doses, compared to pregabalin (8, 9). On the other hand, other studies have shown that pregabalin provides faster and more significant relief of pain compared to gabapentin (10, 11). Regarding long-term safety data for the chronic use of pregabalin and gabapentin, in the case of pregabalin for the treatment of anxiety disorders, good tolerability has been observed with effective disease management (12). Both responders and non-responders showed low and similar discontinuation rates, with a good safety profile across a dosage range of 150–600 mg (12). Another study examined the effects of pregabalin over 2 years with doses above 300 mg for the treatment of patients with partial-onset epilepsy and found a higher discontinuation rate but good disease control (13). The most common but transient adverse events were dizziness, somnolence, headaches, and asthenia (13). In the case of gabapentin, its long-term use in a Japanese population treated with doses between 600 and 1,800 mg for restless legs syndrome showed good disease control; however, there was a 90% rate of adverse events, most of which were mild and transient, with dizziness and somnolence being the most frequent (14). In patients with chronic epilepsy treated for more than 3 years with 1,800 mg of gabapentin, 39% discontinued gabapentin owing to lack of efficacy (15).

It is important to note that there are meta-analyses comparing the efficacy of pregabalin and gabapentin, although direct comparisons between the two drugs have not been conducted. Some of these meta-analyses, such as those conducted by Markman et al. (16) and Mehta et al. (17), have separately evaluated the efficacy of each medication in neuropathic pain. However, there are important aspects that have not been taken into account in the existing reviews, such as treatment costs, optimal drug dosages, and variables related to patients' quality of life and functionality, not just in terms of pain.

In this regard, Ozgencil et al. (18) recommended the conduct of further studies focusing on the dosages of these drugs, aiming to determine the best therapeutic strategy for neuropathic pain. Gammoh et al. (19) highlighted this issue and emphasized the importance of basing therapeutic decisions on available scientific evidence.

Given that both gabapentin and pregabalin are recommended as first-line treatment for chronic neuropathic pain, it is imperative to conduct a direct comparison between these two drugs (20). This comparison will provide more precise information regarding their efficacy and safety, as well as their impact on broader aspects of patients' quality of life and functionality. Patient-reported outcome measures (PROMs) may actually have more health relevance in chronic pain for patients, professionals, and health administrators than the simple assessment of pain intensity (21). In this context, the objective of this meta-analysis is to evaluate and compare pregabalin vs. gabapentin in terms of efficacy and safety in the treatment of neuropathic pain, aiming to provide a solid foundation for clinical decision-making and improve the management of this condition in medical practice.

## 2 Methods

### 2.1 Eligibility criteria

The protocol for this study was registered in PROSPERO (CRD42024565208), and PRISMA guidelines were followed (22). The PICOS search strategy was employed: P: Patients with neuropathic pain (including various pathologies); I: The intervention group comprised patients treated with pregabalin; C: The comparator group consisted of patients treated with gabapentin; O: The primary outcomes assessed were efficacy and safety results; S: The study types included comparative studies (clinical trials and cohort studies).

The exclusion criteria were applied to ensure the quality and relevance of the analysis. Duplicate studies were excluded to prevent the duplication of data and avoid bias. Clinical trial protocols were excluded because they lack complete results and are not suitable for analysis. Studies focused on pediatric patients were excluded to maintain the focus on the intended adult population. Only studies directly comparing pregabalin with gabapentin were included to address the specific research question. Studies that did not share variables were excluded to facilitate meaningful comparisons. Studies with incomplete or missing data were excluded to maintain the integrity and reliability of the analysis. In the case of duplicated studies presenting related information in multiple publications, they were grouped into a single study to avoid duplicating basic characteristics and ensure that each variable was considered only once. Nevertheless, relevant information from each duplicated study was extracted and included in the analysis.

### 2.2 Information sources and search methods for identification of studies

PubMed, Embase, Scopus, and the Cochrane Collaboration Library databases were utilized for this study from March to April 2024. No filters were applied based on publication date or language. In addition to database searches, handsearching was conducted through the references of included studies. Study selection was performed by two authors, and in cases of disagreement, a third author participated to reach a consensus. The search equation employed the following terms: (pregabalin OR Lyrica) AND gabapentin AND neuropathic (Supplementary File 1).

### 2.3 Data extraction and data items

Data extraction was conducted by two authors, and in cases of disagreement, a third author participated to reach a consensus. The baseline characteristics of each article were collected. The variables that were extracted included the Visual Analog Scale (VAS), the percentage of patients with mild or no pain, the number of days with no/mild pain or several pain, the number of days with severe pain, opioid consumption, patient reported outcome measures (including EQ-5D, SF-12/SF-36), quality-adjusted life years (QALYs), total costs, costs per additional test, specialist visits, and adverse events.

Regarding quality of life questionnaires, the EQ-5D is a quality of life scale that assesses five dimensions (mobility, self-care, usual activities, pain/discomfort, and anxiety/depression) with three possible response levels (1–3) (23). The SF-12/SF-36 is a health questionnaire that measures eight dimensions (physical functioning, role limitations due to physical health, bodily pain, general health, vitality, social functioning, role limitations due to emotional problems, and mental health) on a scale from 0 to 100, where higher values indicate better health (24).

### 2.4 Assessment of risk of bias in included studies

For non-randomized studies, the risk of bias was assessed using the Methodological Index for Non-Randomized Studies (MINORS) with a total of 12 items (25). For non-comparative studies, scores ranging from 0 to 4, 5 to 7, 8 to 12, and ≥13 were categorized as very low, low, fair, and high quality, respectively. In comparative studies, scores ranging from 0 to 6, 7 to 10, 11 to 15, and ≥16 were categorized as very low, low, fair, and high quality, respectively (25).

For clinical trials, the risk of bias was evaluated using the Cochrane Review Manager tool. Several domains were considered, including randomization, allocation concealment, blinding of participants and outcome assessors, incomplete outcome data, selective outcome reporting, and other sources of bias. Each domain was assessed as having “low risk of bias,” “uncertain risk of bias,” or “high risk of bias.”

### 2.5 Assessment of results

The statistical analysis was performed using Review Manager 5.4.1 software. For continuous variables, the mean difference or standard mean difference (when studies used different units or scales but were in the same direction) was calculated. For dichotomous variables, odds ratios were calculated. All effect sizes were reported with 95% confidence intervals. To assess heterogeneity among studies, the *χ*^2^ test and *I*^2^ statistic were used. *I*^2^ values greater than 25%, 50%, and 75% indicated low, moderate, and high heterogeneity, respectively. If there was no significant heterogeneity, a fixed-effects model was employed. In the presence of significant heterogeneity, a random-effects model was utilized. Variables with insufficient data for meta-analysis were summarized qualitatively in a narrative synthesis. Precise data points from study figures were extracted using WebPlotDigitizer software version 4.5. Missing data were managed according to the guidelines outlined in the Cochrane Handbook (26).

### 2.6 Risk of bias across the studies

Publication bias was assessed through visual inspection of funnel plots. Review Manager 5.4.1 software was used for this analysis. Funnel plots typically display the standard error on the *y*-axis and the effect size on the *x*-axis. By visually examining the symmetry of the funnel plot, potential publication bias can be identified. Asymmetry in the funnel plot may indicate the presence of publication bias, with smaller or non-significant studies potentially being underrepresented.

### 2.7 Additional analyses

Subgroup analysis was conducted based on the duration of follow-up for variables that were divided according to the results.

A sensitivity analysis was conducted by excluding the study with the highest weight from the analysis. This approach aimed to assess the robustness of the findings and evaluate the potential impact of the study on the overall results. Sensitivity analysis was also performed based on the dosage of the medications. For pregabalin, low doses were defined as ≤300 mg (PL), and high doses were defined as >300 mg (PH) (27). For gabapentin, low doses were defined as ≤1,800 mg (GL), and high doses were defined as >1,800 mg (GH) (28).

Furthermore, the GRADE (Grading of Recommendations Assessment, Development, and Evaluation) approach was utilized. GRADE assesses the quality of evidence and provides a framework for evaluating the certainty of the findings. It takes into account factors such as study design, risk of bias, inconsistency, indirectness, imprecision, and publication bias (29).

## 3 Results

### 3.1 Study selection

**Figure 1:**
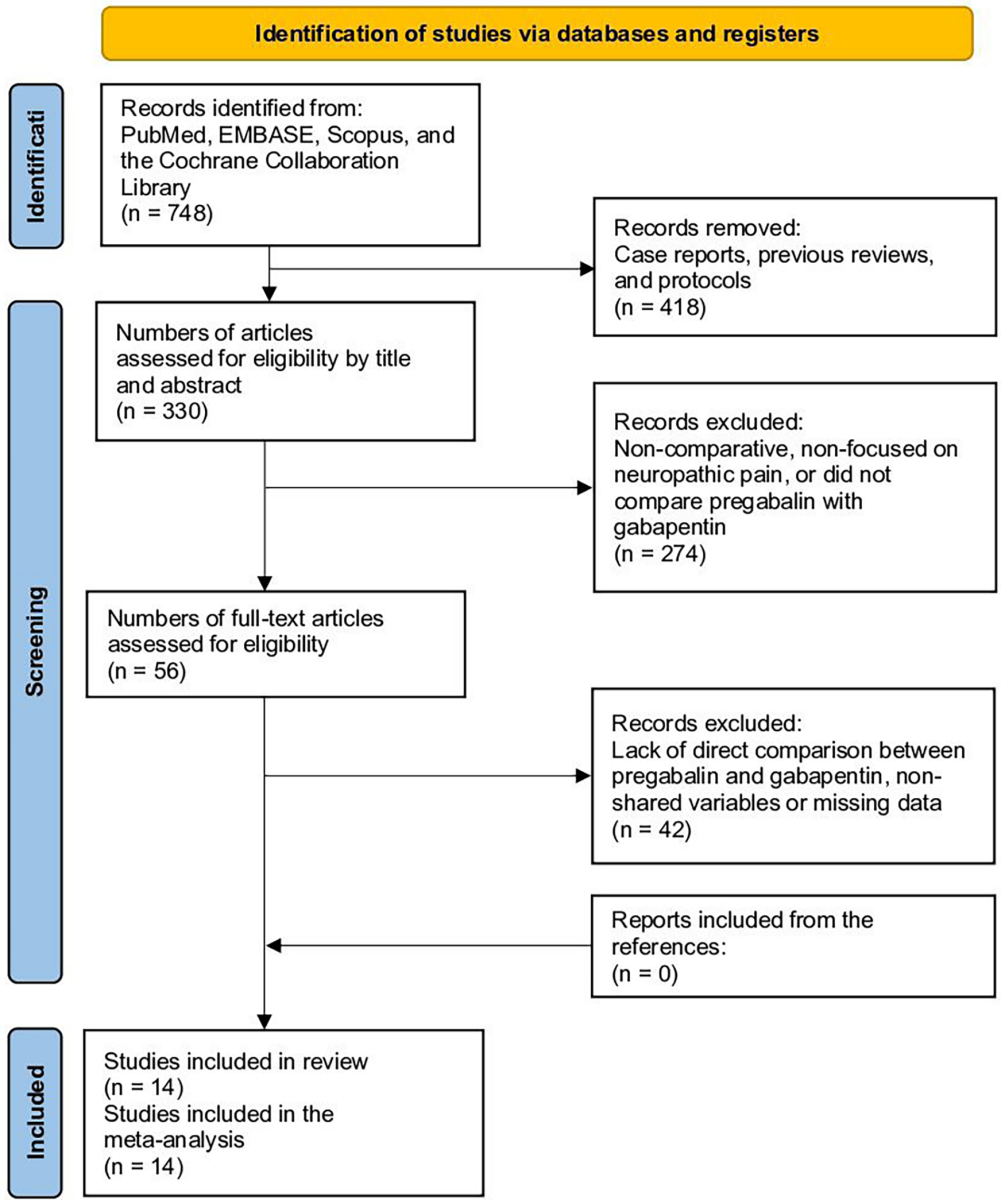
Study selection flow diagram (preferred reporting items for systematic reviews and meta-analyses).

A total of 748 studies were obtained after searching the databases. After filtering for comparative studies and excluding case reports, previous reviews, and protocols, 330 studies remained, eliminating 418. Upon reviewing titles and abstracts, 274 studies were excluded as they were non-comparative, not focused on neuropathic pain, or did not compare pregabalin with gabapentin, resulting in 56 studies. Among the 56 studies, after reviewing the full text, 42 were eliminated due to the lack of direct comparison between pregabalin and gabapentin, unshared variables, or missing data, resulting in 14 studies. No additional studies were added through reference review. Finally, 14 studies were included in the meta-analysis (Figure 1) (8–11, 18, 19, 30–37), 27–34.

### 3.2 Study characteristics

Table 1 presents the baseline characteristics of the included studies. A total of 14 studies and 3,346 patients were included (1,714 in the pregabalin group and 1,632 in the gabapentin group). The age range in the pregabalin group varied from 32.0 to 61.9 years, while in the gabapentin group, it ranged from 36.0 to 61.9 years. The number of males, etiology, and doses of pregabalin and gabapentin are displayed in Table 1.

**Table 1:** Baseline characteristics of the included studies.

Study	Region	Type of study	Follow-up (weeks)	n Pregabalina/Gabpentin	Age Pregabalin/Gabpentin	Male Pregabalin/Gabpentin	Etiology	Doses Pregabalina/Gabpentin (mg/day)
Agarwal et al. (8)	United Arab Emirates	Retrospective cohort	NR	54/72	32.0/36.0	54/72	Urological chronic pelvic neuropathic pain syndrome	150/400
Atalay et al. (30)	Turkey	RCT	14.0	25/25	NR/NR	NR/NR	Peripheral neuropathy	75/300
Athanasakis et al. (31)	Greece	Retrospective cohort	12.0	141/193	NR/NR	NR/NR	PHN and DPN	457/2,400
Devi et al. (10)	United Arab Emirates	RCT	12.0	52/50	55.4/57.2	32/35	DPN	300/1,800
Gammoh et al. (19)	Jordan	RCT	6.0	28/36	NR/NR	oct-16	Neuropathic low back pain	300/800
Gore et al. (32)	USA	Retrospective cohort	26.0	100/151	52.8/55.8	38/65	PHN	199/823
Irving et al. (33)	USA	RCT	12.0	134/135	61.9/61.9	76/83	DPN	300/≥900
Mishra et al. (34)	India	RCT	4.0	30/30	NR/NR	NR/NR	Neuropathic cancer pain	600/1,800
Ozgencil et al. (18)	Turkey	RCT	NR	30/30	51.9/50.6	dic-15	Neuropathic spine postoperative pain	300/1,200
Pérez et al. (11)	Spain	Pospective cohort	12.0	88/44	59.4/58.5	45/24	DPN, PHN, or trigeminal neuralgia	202/1,263
Rauck et al. (9)	UK, USA	RCT	20.0	66/234	57.7/58.6	34/142	DPN	300/1,200–2,400–3,600
Rodríguez et al. (35)	Spain	Retrospective cohort	12.0	141/193	NR/NR	NR/NR	Diabetic polyneuropathy or PHN	457/2,400
Sicras-Mainar et al. (36)	Spain	Retrospective cohort	NR	764/399	59.8/58.1	271/169	Peripheral neuropathy	75–600/<900->1,800
Toth et al. (37)	Canada	Retrospective cohort	52.0	61/40	55.9/58.4	30/13	Peripheral neuropathy	389–405/2,235

DPN, diabetic peripheral neuropathy; NR, not reported; PHN, post-herpetic neuralgia; RCT, randomized clinical trial.

### 3.3 Risk of bias

Regarding the randomized studies, they exhibited a moderate risk of bias (Supplementary Figure 1; Supplementary File 2). Specifically, the majority of studies lacked patient and evaluator blinding. Among the non-randomized studies, 5 out of 7 demonstrated high quality, while 2 out of 7 had acceptable quality (Table 2). These studies were deficient in prospective data collection and reporting patient attrition at the end of the follow-up period.

**Table 2:** Assessment of the quality of studies through methodological index for non-randomized studies (MINORS).

Study	Clearly stated aim	Consecutive patients	Prospective collection data	Endpoints	Assessment endpoint	Follow-up period	Loss less than 5%	Study size	Adequate control group	Contemporary group	Baseline control	Statistical analyses	MINORS
Agarwal et al. (8)	2	2	0	1	1	0	0	2	2	2	2	2	16
Athanasakis et al. (31)	2	0	0	2	2	2	0	2	0	0	0	2	12
Gore et al. (32)	2	2	0	2	2	2	0	2	2	2	1	2	19
Pérez et al. (11)	2	2	2	2	2	2	0	2	2	2	2	2	22
Rodríguez et al. (35)	2	0	0	2	2	2	0	2	0	0	0	2	12
Sicras-Mainar et al. (36)	2	2	0	2	2	0	0	2	2	2	2	2	18
Toth et al. (37)	2	2	2	2	2	2	0	2	2	2	2	0	20

### 3.4 Patient reported outcome measures (PROMs)

**Figure 2:**
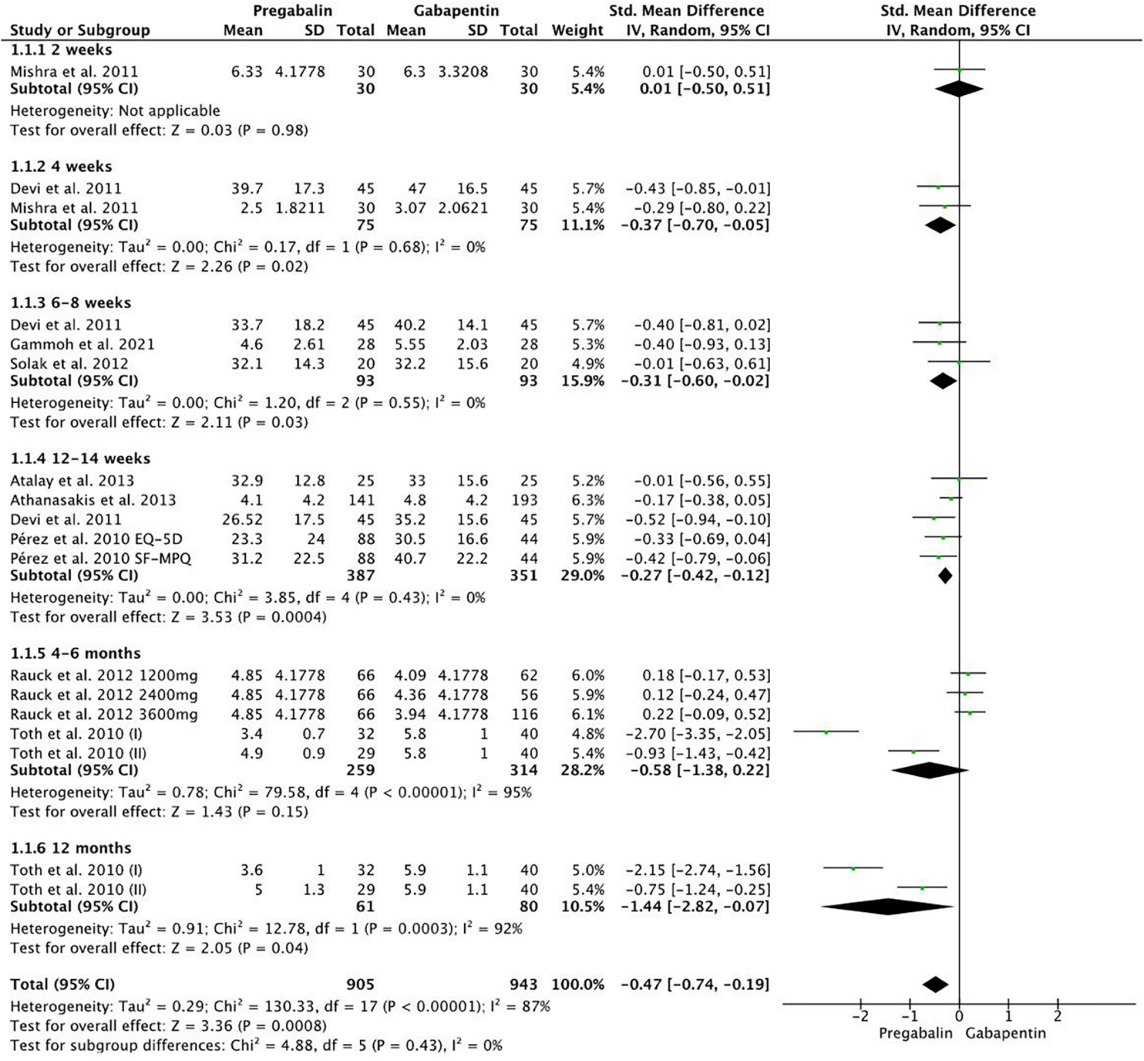
Forest plot showing significant differences in favor of pregabalin compared to VAS.

The global VAS (Visual Analog Scale) showed significantly better results in favor of pregabalin (SMD −0.47, 95% CI −0.74 to −0.19; participants = 1,848; studies = 9; I2 = 87%) (Figure 2). At two weeks, there were no significant differences (SMD 0.01, 95% CI −0.50 to 0.51; participants = 60; studies = 1; *I*^2^ = 0%). At 4 weeks (SMD −0.37, 95% CI −0.70 to −0.05; participants = 150; studies = 2; *I*^2^ = 0%), 6–8 weeks (SMD −0.31, 95% CI −0.60 to −0.02; participants = 186; studies = 3; I2 = 0%), and 12–14 weeks (SMD −0.27, 95% CI −0.42 to −0.12; participants = 738; studies = 4; *I*^2^ = 0%), the pregabalin group showed significant pain improvement compared to gabapentin. At 4–6 months, no significant differences were found (SMD −0.58, 95% CI −1.38 to 0.22; participants = 573; studies = 2; I2 = 95%), while at 12 months, there were significant differences in favor of pregabalin (SMD −1.44, 95% CI −2.82 to −0.07; participants = 141; studies = 1; *I*^2^ = 92%).

**Figure 3:**
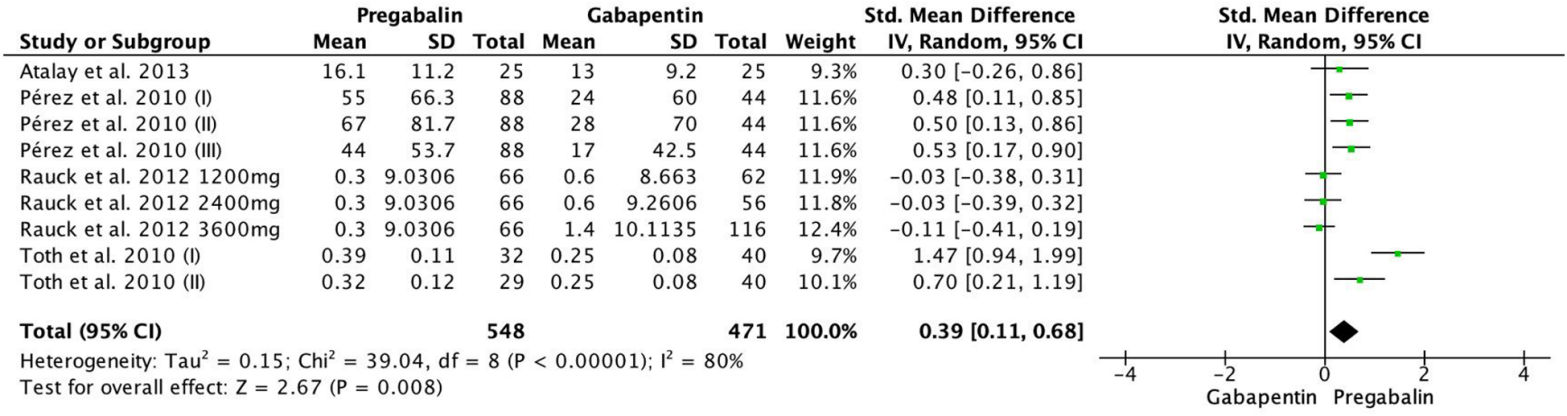
Forest plot illustrating the change in SF-12/SF-36/EQ-5D, which was significantly greater in the pregabalin group than in the gabapentin group.

The change in SF-12/SF-36/EQ-5D was significantly greater in the pregabalin group compared to gabapentin (SMD 0.39, 95% CI 0.11–0.68; participants = 1,019; studies = 4; *I*^2^ = 80%) (Figure 3).

There were no significant differences in the percentage of patients with no/mild pain (OR 0.71, 95% CI 0.32–1.59; participants = 1,024; studies = 6; *I*^2^ = 86%). The number of days with no/mild pain was significantly higher in the pregabalin group (MD 9.00, 95% CI 8.93–9.07; participants = 466; studies = 2; *I*^2^ = 0%). Additionally, the number of days with severe pain was significantly lower in the pregabalin group (MD −3.00, 95% CI −4.96 to −1.04; participants = 466; studies = 2; *I*^2^ = 100%).

### 3.5 Opioid consumption

**Figure 4:**
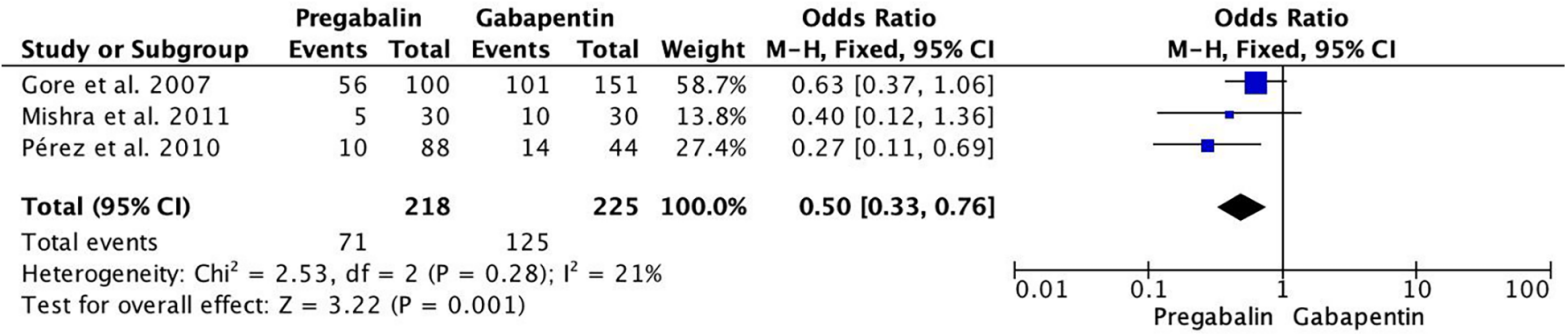
Forest plot demonstrating opioid consumption. Pregabalin was associated with significantly lower opioid consumption than gabapentin (OR 0.50, 95% CI 0.33 0.76).

Regarding opioid consumption, it was significantly lower in the pregabalin group (OR 0.50, 95% CI 0.33–0.76; participants = 443; studies = 3; *I*^2^ = 21%) (Figure 4).

### 3.6 Cost analyses

**Figure 5:**
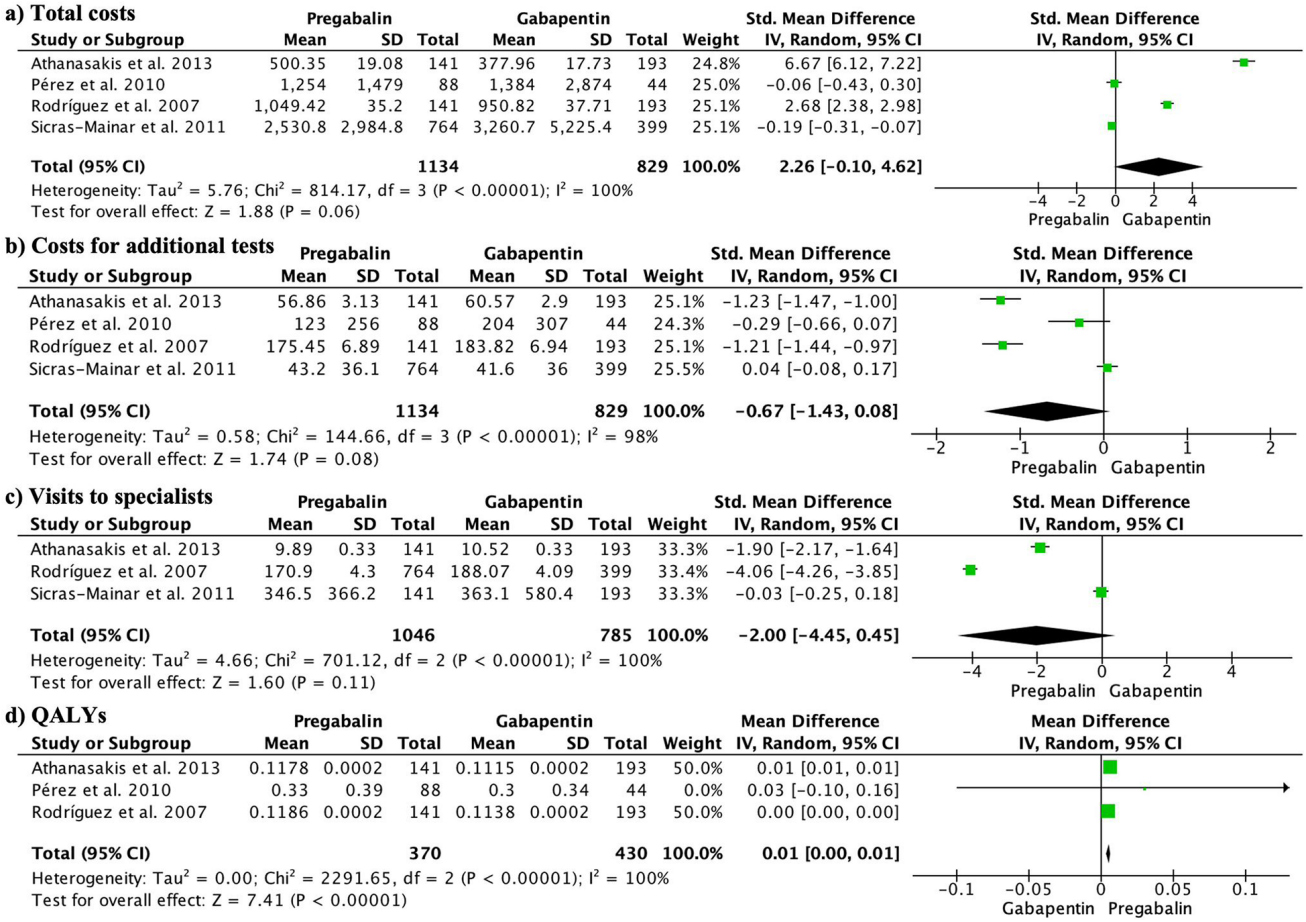
Forest plots presenting the economic analysis. There were no significant differences in total costs **(5a)**, cost per additional test **(5b)**, or mean number of specialist visits **(5c)**. However, QALYs significantly favored the pregabalin group **(5d)**.

The total costs did not show significant differences between the groups (SMD 2.26, 95% CI −0.10 to 4.62; participants = 1963; studies = 4; *I*^2^ = 100%) (Figure 5a). The cost per additional test also did not exhibit significant differences (SMD −0.67, 95% CI −1.43 to 0.08; participants = 1,963; studies = 4; *I*^2^ = 98%) (Figure 5b). Furthermore, there were no significant differences in the mean number of specialist visits (SMD −2.00, 95% CI −4.45 to 0.45; participants = 1,831; studies = 3; *I*^2^ = 100%) (Figure 5c). On the other hand, the QALYs significantly favored the pregabalin group (MD 0.01, 95% CI 0.00–0.01; participants = 800; studies = 3; *I*^2^ = 100%) (Figure 5d).

### 3.7 Adverse events

Adverse events are presented in Table 3. Overall, there were no significant differences in terms of total adverse events. However, when considering specific adverse events, gabapentin showed a higher incidence of nausea and vomiting compared to pregabalin. There were no significant differences in the occurrence of other specific adverse events.

**Table 3:** Adverse events.

Effect size	*n* studies	*n* participants	Fixed effect model (OR 95% CI)	*I*^2^ (%)	*P*-value
Any adverse event	7	944	OR 1.15, 95% CI 0.67–1.97^a^	65	0.61
Nausea	7	943	OR 0.36, 95% CI 0.20–0.63	0	0.0004
Vomiting	6	863	OR 0.33, 95% CI 0.13–0.85	0	0.02
Insomnia	2	349	OR 0.63, 95% CI 0.16–2.46	19	0.51
Edema	4	842	OR 2.80, 95% CI 0.84–9.30^a^	69	0.09
Dizziness	6	815	OR 1.07, 95% CI 0.71–1.61	0	0.76
Somnolence	8	815	OR 1.24, 95% CI 0.83–1.87	38	0.29
Dry mouth	6	653	OR 1.15, 95% CI 0.52–2.54	17	0.73
Diarrhea	4	512	OR 1.26, 95% CI 0.60–2.65	0	0.54
Constipation	4	512	OR 1.56, 95% CI 0.76–3.22	0	0.23
Headache	6	633	OR 1.66, 95% CI 0.92–3.01	0	0.09

^a^ Random effect model.

### 3.8 Additional analyses

**Figure 6:**
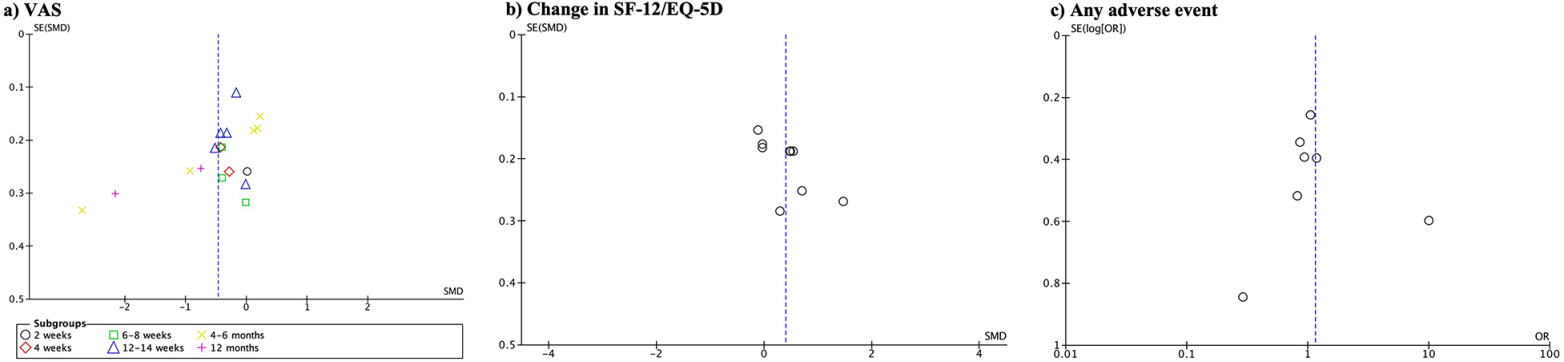
Funnel plot showing evidence of publication bias observed for VAS **(a)**, change in SF-12/SF-36/EQ-5D **(b)**, and adverse events **(c)**.

Publication bias is depicted in Figure 6. There was publication bias observed for VAS, change in SF-12/SF-36/EQ-5D, and adverse events.

The sensitivity analysis, eliminating the study with the highest weight, did not change the direction of the results except for the cost for additional test, being significantly lower in the pregabalin group compared to gabapentin (SMD −0.93, 95% CI −1.44 to −0.43; participants = 800; studies = 4; *I*^2^ = 90%) (Supplementary Figure 2). Similarly, when sensitivity analysis was conducted by excluding the study by Rauck et al. (9) because it used gabapentin enacarbil, which is an extended-release form and thus different from the rest of the studies, the Visual Analog Scale (VAS) showed significant differences in favor of pregabalin at 4–6 months (SMD −1.80, 95% CI −3.54, −0.07; participants, 141; studies, 3; *I*^2^ = 94%).

The sensitivity analysis considering the doses of pregabalin and gabapentin is presented in Table 4. The VAS analysis showed favorable results for both PH vs. GH and PL vs. GL in favor of pregabalin. Opioid consumption could only be compared between PL vs. GL, demonstrating significant differences in favor of low-dose pregabalin. Regarding the change in SF-12/SF-36/EQ-5D, PH vs. GH and PL vs. GL showed significant differences in favor of pregabalin. In terms of any adverse events, the PL vs. GL group, specifically low-dose pregabalin, exhibited a lower incidence of complications compared to gabapentin. Nausea and vomiting, when comparing PL vs. GL, continued to show higher incidence in the gabapentin group. There were no significant differences in other complications.

**Table 4:** Outcomes depending on the doses.

Effect size	*n* studies	*n* participants	Fixed effect model (OR 95% CI)	*I*^2^ (%)
VAS				
PH vs. GH	6	661	SMD −1.31, 95% CI −2.23 to −0.39^a^	95
PL vs. GL	7	622	SMD −0.21, 95% CI −0.41 to −0.01	34
% No/mild pain				
PL vs. GL	3	386	OR 0.57, 95% CI 0.14–2.38^a^	90
*N* opioid consumption				
PL vs. GL	2	383	OR 0.52, 95% CI 0.33–0.81	58
Change SF-12/SF-36/EQ5D				
PH vs. GH	2	141	SMD 1.08, 95% CI 0.33–1.83^a^	77
PL vs. GL	5	574	SMD 0.35, 95% CI 0.13–0.58	41
PL vs. GH	2	304	SMD −0.08, 95% CI −0.31 to 0.15	0
Any adverse event				
PL vs. GL	3	499	MD 0.30, 95% CI 0.09–0.51	6
Nausea				
PL vs. GL	5	639	OR 0.27, 95% CI 0.13–0.55	1
Vomiting				
PL vs. GL	5	681	OR 0.33, 95% CI 0.12–0.89	0
Dizziness				
PH vs. GH	2	895	OR 1.22, 95% CI 0.83–1.80	30
PL vs. GL	4	370	OR 0.92, 95% CI 0.50–1.70	0
PL vs. GH	2	304	OR 0.97, 95% CI 0.50–1.89	0
Headache				
PH vs. GH	2	141	OR 2.87, 95% CI 0.82–10.03	0
PL vs. GL	2	188	OR 0.96, 95% CI 0.35–2.68^a^	55
PL vs. GH	2	304	OR 1.90, 95% CI 0.75–4.80	0
Somnolence				
PH vs. GH	2	131	OR 1.28, 95% CI 0.61–2.70^a^	86
PL vs. GL	2	370	OR 1.33, 95% CI 0.66–2.68	26
PL vs. GH	2	304	OR 1.13, 95% CI 0.57–2.24	
Dry mouth				
PH vs. GH	2	141	OR 2.87, 95% CI 0.82–10.03	0
PL vs. GL	2	208	OR 0.69, 95% CI 0.13–3.60	16
PL vs. GH	2	304	OR 0.43, 95% CI 0.08–2.14	31
Diarrhea				
PL vs. GL	2	208	OR 0.80, 95% CI 0.25–2.58^a^	56
Constipation				
PL vs. GL	2	208	OR 1.14, 95% CI 0.35–3.66	46

^a^ Random effect model; GL, gabapentin low doses; PL, pregabalin low doses.

The GRADE assessment is presented in Table 5. The evidence was moderate for the VAS variable and the number of patients with opioid consumption. It was of low certainty for the change in SF-12/SF-36/EQ-5D variable and very low for the rest of the variables.

**Table 5:** GRADE assessment of the quality of the evidence and the strength of the recommendations.

Certainty assessment							No. of patients		Effect		Certainty	Importance
No. of studies	Study design	Risk of bias	Inconsistency	Indirectness	Imprecision	Other considerations	[Intervention]	[Comparison]	Relative (95% CI)	Absolute (95% CI)		
VAS												
13	Randomised trials	Not serious	Not serious	Serious^a^	Not serious	Publication bias strongly suspected dose response gradient^b^	905	943	–	SMD 0.47 lower (0.74 lower to 0.19 lower)	⊕⊕⊕◯ moderate	Critical
% No/mild pain												
6	Non-randomised studies	Not serious	Serious^c^	Serious^a^	Not serious	Publication bias strongly suspected^b^	144/481 (29.9%)	172/543 (31.7%)	OR 0.71 (0.32 to 1.59)	69 fewer per 1000 (from 188 fewer to 108 more)	⊕◯◯◯ very low	CriticaL
Days with no/mild pain												
2	Non-randomised studies	Not serious	Not serious	Serious^a^	Not serious	None	229	237	–	MD 9 higher (8.93 higher to 9.07 higher)	⊕◯◯◯ very low	Critical
*n* opioid conumption												
3	Non-randomised studies	Not serious	Not serious	Serious^a^	Not serious	Strong association dose response gradient	71/218 (32.6%)	125/225 (55.6%)	OR 0.50 (0.33 to 0.76)	171 fewer per 1000 (from 264 fewer to 68 fewer)	⊕⊕⊕◯ moderate	Critical
Change SF-12/SF-36/EQ-5D												
9	Non-randomised studies	Not serious	Not serious	Serious^a^	Not serious	Publication bias strongly suspected strong association dose response gradient^b^	548	471	–	SMD 0.39 higher (0.11 higher to 0.68 higher)	⊕⊕◯◯ low	Critical
Any												
7	Randomised trials	Not serious	Serious^c^	Serious^a^	Not serious	Publication bias strongly suspected^b^	265/445 (59.6%)	289/499 (57.9%)	OR 1.15 (0.67 to 1.97)	34 more per 1,000 (from 99 fewer to 151 more)	⊕◯◯◯ very low	Important

CI, confidence interval; MD, mean difference; OR, odds ratio; SMD, standardised mean difference.

^a^ Different etiologies, doses, study design and follow-up times.

^b^ Publication bias assessed by visual inspection of funnel plots.

^c^ The results showed a wide variability.

## 4 Discussion

The study compared the effectiveness and safety of pregabalin and gabapentin in neuropathic pain management. Pregabalin showed significantly better results than gabapentin in terms of pain reduction, as measured by the Visual Analog Scale (VAS). The change in SF-12/SF-36/EQ-5D, indicating improvement in quality of life, was also significantly greater in the pregabalin group. Pregabalin was associated with lower opioid consumption and a lower incidence of adverse events, particularly nausea and vomiting. Also, the cost-effectiveness measured by QALYs of pregablin was significantly more favorable than gabapentin. However, there were no significant differences in overall adverse events. Sensitivity analysis considering different doses confirmed the superiority of pregabalin.

Pregabalin demonstrated significant improvement in pain assessed using the VAS scale and in functionality measured by the SF-12/SF-36 and EQ-5D. One possible explanation for why pregabalin outperformed gabapentin in terms of pain and quality of life/functionality could be its efficacy in important aspects such as anxiety, depression, and sleep disorders (38, 39). In fact, it is important for medications to address multiple aspects since neuropathic pain is associated with other conditions like anxiety, depression, and sleep disorders (40). Therefore, pregabalin has shown effectiveness in anxiety and insomnia, even at doses lower than 300 mg (33). Additionally, in patients with fibromyalgia and depression, pregabalin improved scales related to depression and pain (41). Furthermore, pregabalin demonstrated significant improvement from 4 weeks onwards, except at 12–14 weeks. This could be attributed to the specific study at that time point, (9), which did not show significant differences with any of the gabapentin doses, although the trend favored gabapentin. Additionally, it is worth noting that Rauck et al. (9) utilized gabapentin enacarbil, an extended-release formulation, which differs from other studies. It is important to consider that the variables of functionality and quality of life, in addition to being influenced by various factors and utilizing the standard mean difference, were reported in only four studies. Several studies included in these outcomes originated from the same research group. A sensitivity analysis was conducted to examine the impact of different combinations by retaining only one study from each group, even with varying dosages, and the trend largely remained in favor of pregabalin, albeit to a lesser extent than that indicated by the global forest plot. This may also be because of the limited number of articles included in the analysis.

Pregabalin has proven to be an effective option in reducing pain, especially during acute pain episodes (42), due to its rapid action and effective mechanisms on seizures (43). This characteristic could be beneficial in patients with neuropathic pain related to cancer, as they experience both baseline pain and pain spikes (34). Furthermore, pregabalin has shown to reduce opioid doses and the adverse effects associated with their use (44). Clinical trials that have independently analyzed pregabalin have yielded positive results, especially with higher doses (up to 600 mg daily), demonstrating its effectiveness in pain reduction (44). Additionally, combining tricyclic antidepressants or gabapentinoids with opioids has been found to improve neuropathic pain in both cancer and non-cancer patients (44). It is important to note that when combining gabapentin with opioids, dose adjustments of gabapentin may be necessary due to delayed renal elimination (44).

In addition to its clinical effectiveness, research has investigated how pregabalin affects the brains of patients with chronic pain, revealing that it reduces the levels of certain chemicals in the brain region associated with pain processing (45). This reduction is associated with changes in connectivity between different brain regions involved in the experience of chronic pain. These findings suggest that pregabalin's ability to modulate these neurochemical and connectivity changes may be one of the reasons for its efficacy in treating neuropathic pain (45). Furthermore, baseline levels of glutamate and connectivity of the insula cortex may be predictors of the analgesic response to pregabalin, supporting the idea of a more personalized approach in chronic pain treatment (45).

Pregabalin and gabapentin are medications that have some differences in their mechanism of action. Both act on voltage-dependent calcium channels in presynaptic neurons, reducing the release of excitatory neurotransmitters such as glutamate, thereby decreasing the transmission of pain signals in the central nervous system (46). However, pregabalin has a higher affinity for these channels, resulting in more potent inhibition of neurotransmitter release. Additionally, it selectively binds to the α2δ-1 subunit of calcium channels in the central nervous system, contributing to its analgesic effect and reducing the release of other neurotransmitters (46).

Neuropathic pain is often associated with chronic conditions, so it is important to approach the patient in a multidisciplinary manner, taking into account different physical and psychological components to achieve a greater impact on their quality of life. In addition to pain management, in cases of diabetic neuropathy, it is crucial to recommend proper glucose control, as poor control can increase nerve damage and, consequently, neuropathic pain. Fortunately, it has been observed that pregabalin does not affect glucose levels or hemoglobin A1c in patients with painful diabetic neuropathy (47). Beneficial effects of pregabalin have even been observed in endotoxin-induced pancreatic pathology in elderly rats (48). On the other hand, gabapentin has been found to affect glucose levels in certain contexts. In a study conducted in cats, it was found that the use of gabapentin before intradermal tests increased glucose concentrations compared to the untreated group (49). However, cases of gabapentin-induced hypoglycemia in patients have also been reported (47). Two possible mechanisms are proposed to explain this hypoglycemia. One of them is related to the activation of GABA receptors in pancreatic beta cells, while the other involves L-type calcium channels, specifically the alpha2delta-2 subunit, present in the pancreas (50).

When it comes to the possibility of developing tolerance to pregabalin and gabapentin, studies show that gabapentinoids usually do not cause the same quick development of tolerance as direct agonists like opioids (51). This trait is especially important for treating chronic pain, as long-term pharmaceutical effectiveness is critical for patient outcomes. The analgesic effects of pregabalin and gabapentin are facilitated by their modes of action, which involve binding to the α2δ subunit of voltage-activated calcium channels. Additionally, gabapentinoids have been shown to reduce analgesic tolerance and opioid-induced hyperalgesia (52). When combined with opioids, gabapentin's capacity to control glutamatergic input through NMDA receptors enhances its overall analgesic effect, which is responsible for this protective effect (52). Although it might not be as noticeable as it is with opioids, the development of tolerance to gabapentinoids is still present. According to a previous study, some patient groups may continue to lose effectiveness over time (53).

When analyzing different comparisons and combinations of pregabalin with other drugs for the relief of neuropathic pain, interesting results have been obtained. Firstly, the comparison between duloxetine and pregabalin did not show significant differences in pain reduction at 24 h, but patients had a preference for pregabalin in terms of overall impression and experienced fewer dizziness side effects (54). On the other hand, the combination of imipramine and pregabalin proved to be more effective in pain relief for patients with painful polyneuropathy, although a higher dropout rate and more side effects were observed (55). In the COMBO-DN study, the combination of duloxetine and pregabalin was evaluated in patients with diabetic peripheral neuropathic pain, and favorable results were found in terms of efficacy and safety. It is important to note that duloxetine showed better analgesia compared to pregabalin when administered at half of its maximum dose (56). Within the high-dose mono- therapy group, 46.9% of patients treated with 600 mg/day pregabalin experienced a pain reduction of P50% compared to 28.4% treated with 120 mg/day duloxetine (56). Furthermore, the combination of amitriptyline with pregabalin has been supported for the treatment of diabetic peripheral neuropathy (57). However, in the case of gabapentin, it is not considered an approved option for diabetic peripheral neuropathic pain and is generally combined with duloxetine (33). Nevertheless, gabapentin has been observed to have a synergistic effect in reducing pain when combined with opioids (58). It is important to note that this combination may carry side effects such as a higher risk of constipation compared to gabapentin alone, as well as a higher risk of dry mouth compared to morphine (58). Therefore, the selection of the appropriate combination should be made considering the benefits and potential associated side effects.

This meta-analysis did not find significant differences in overall complications, possibly due to the use of low doses of the drugs in most studies. However, a higher incidence of nausea and vomiting was observed with gabapentin compared to pregabalin. According to the Canadian Pain Society, both medications are considered first-line treatments for chronic neuropathic pain, but guidelines caution about potential adverse effects such as drowsiness, dizziness, peripheral edema, and blurred vision (20). Our study revealed that gabapentin had an almost three-fold higher risk of causing nausea and vomiting compared to pregabalin. It is important to consider the recommended doses, such as 100–300 mg/day for gabapentin and 25–150 mg/day for pregabalin, according to the Canadian Society (20).

Regarding the long-term treatment adherence impacted by the side effect profiles of pregabalin and gabapentin, Stacey et al. evaluated the effects of pregabalin on refractory neuropathic pain over a 15-month period, with treatment administered in 3-month intervals followed by 3- to 28-day “drug holidays” (59). The most common adverse events reported were somnolence (22%) and dizziness (19%), with discontinuation due to adverse events (12.3%) and a lack of effectiveness (6.2%). In another 52-week randomized controlled trial with placebo, Satoh et al. confirmed the long-term mild adverse effects of pregabalin on both diabetic neuropathy and postherpetic neuralgia (60). Similarly, Ogawa et al. reported the impact of gabapentin in a study of patients with spinal cord injuries followed for up to 36 months, with a discontinuation rate of 22% due to adverse events (61). Putzke et al. also contributed to the understanding of gabapentin safety through a long-term study that compared patients prescribed the drug for at least 60 days with an unexposed group from 2002 to 2015 (62). This study analyzed the incidence of falls, fractures, and altered mental status over two years, highlighting a dose-response relationship where higher risks were observed at doses above 600 mg/day (63).

Another important point to consider is drug consumption, which is a topic of great interest. It has been observed that pregabalin has significantly lower opioid consumption compared to gabapentin. This difference is relevant in the context of the opioid epidemic, which has reached alarming proportions due to widespread abuse of prescription opioids and the rise of illicit opioids (64). In 2015, over 33,000 deaths were attributed to opioid overdoses, highlighting the need to find effective alternatives to control and eradicate this devastating crisis (27). In this regard, it is crucial to focus on multimodal analgesia management and explore new drugs as part of a comprehensive strategy. For future studies, it is recommended to further examine the causes or scenarios that lead to the prescription of additional medication in order to gain a more comprehensive understanding of the factors influencing opioid consumption (28).

In our study, we found that only four studies provided information related to costs. No significant differences were found in total costs, costs of additional tests, or specialist consultations. However, quality-adjusted life years were significantly higher with pregabalin. These findings are supported by existing literature, which indicates that pregabalin is more costly but also more effective than gabapentin in pain treatment. Although pregabalin carries an additional cost, the clinical benefits in terms of reduced resource utilization and improved patient outcomes partially offset this additional cost. Overall, pregabalin is considered to have a better balance between cost and effectiveness (28). Other studies have also demonstrated that both generic and branded forms of pregabalin are highly cost-effective compared to placebo (27). Therefore, including generic pregabalin in the reimbursement list would be a more rational option, considering its similar efficacy to the branded version but at a lower cost (27). However, it is important to consider the limitations in the number of available studies to evaluate the influence of dosage on cost-effectiveness relationship. From the perspective of the Spanish healthcare system, it has been concluded that pregabalin is more cost-effective than gabapentin in the majority of studied cases, implying additional health benefits and a positive impact on patients' work capacity (32).

Although this meta-analysis compared the mentioned outcomes, there were variables that could not be included in the meta-analysis but were reported in individual studies. For instance, Solak et al. (65) observed that pregabalin improved pruritus to a greater extent than gabapentin in patients with uremic pruritus in hemodialysis patients with documented peripheral neuropathy. Pérez et al. found that pregabalin reduced symptoms of depression and anxiety in patients with peripheral neuropathy, although no significant differences were found (11). However, Ozgencil demonstrated a significant reduction in anxiety in patients treated with pregabalin (18). Ozgenzil et al. (18) also found a higher percentage of patient satisfaction in the pregabalin group compared to gabapentin. These variables are of great interest and should be considered in future studies, ensuring homogeneous reporting to establish robust and reliable results.

When comparing different clinical guidelines, there are varied recommendations from the international directives. French guidelines recommend pregabalin as a second-line treatment after gabapentin, highlighting a preference based on a traditional escalation approach (66). In contrast, the Spanish and Canadian guidelines place gabapentin and pregabalin on equal footing but emphasize the complexities associated with dosing adjustments for gabapentin (67). Our meta-analysis provides crucial data for this debate by suggesting an advantage of pregabalin over gabapentin in the treatment of neuropathic pain, especially in cases where opioid sparing is crucial.

This study has several limitations that should be considered. First, not all studies included in the analysis were randomized, which introduces a potential bias. Furthermore, the number of studies with adequate blinding is limited. Despite the relatively large number of studies (14 studies), the low number of studies on shared variables hindered subgroup analysis and consideration of different confounding factors. Future studies should prioritize the analysis of Minimal Clinically Important Difference (MCID) to assess clinical relevance. Additionally, the use of multiple scales and limited homogeneity among them make it challenging to compare results and explore different perspectives, such as psychological aspects or specific measures of neuropathic pain. The inclusion of various neuropathic pain etiologies, such as diabetic peripheral neuropathy, postherpetic neuralgia, back pain or urologic chronic pain, adds complexity owing to inherent differences between conditions. The specific type of opioid used for opioid consumption has not yet been specified. Furthermore, it was not possible to individually assess the characteristic symptoms and signs of neuropathic pain, such as allodynia, hyperalgesia, and burning pain, owing to inconsistencies in extracting these data from the original studies. Moreover, incomplete data reporting in some studies necessitated the use of the Cochrane rules to estimate standard deviations, as well as studies that reported results through various subgroups rather than providing a consolidated overall outcome. Finally, substantial statistical heterogeneity was observed across various outcomes, notably in global VAS, quality of life scales, and particularly in cost-related outcomes, such as cost per additional test, number of specialist visits, and QALYs. This heterogeneity stems from differences in the study design, population characteristics, and measurement techniques across the included studies. Given this variability, caution should be exercised when interpreting the results. It is essential to consider the diverse contexts and methodologies that contribute to these variations when applying our findings to clinical practice or policy making. To address these limitations, future research should emphasize randomized controlled trials, increase the number of studies for each shared variable, enhance scale homogeneity, focus on specific neuropathic pain conditions, and ensure comprehensive reporting of data.

This study, on the other hand, has several strengths that contribute to its robustness. First, it is the most recent and comprehensive meta-analysis to date that has incorporated a substantial number of articles. Furthermore, it encompasses a wide range of variables, including efficacy measured using different pain scales, quality of life, opioid consumption, adverse events, and costs. Such a comprehensive inclusion of variables provides practical information that can be directly applied to daily clinical practice. Moreover, meticulous consideration was given to the dosages in all possible measurements, and extensive analyses were conducted to control for heterogeneity and comply with the established standards.

## 5 Conclusion

In conclusion, the findings of this study support that pregabalin provides substantial advantages over gabapentin in the management of neuropathic pain. Patient-reported outcome measures, such as the Visual Analog Scale (VAS) and SF-12/SF-36/EQ-5D, consistently demonstrated pain improvement and greater improvement in quality of life in the pregabalin group. Furthermore, the pregabalin group exhibited lower opioid consumption, which may indicate its potential as an alternative or adjunct to opioids in pain management. Adverse events analysis revealed a higher incidence of nausea and vomiting in the gabapentin group. Physicians may consider the potential benefits of pregabalin based on these results, but individual patient characteristics and preferences should also be taken into account when making treatment decisions. These findings serve as a valuable reference for future research, guideline development, and clinical decision-making in the field of pain management, advancing our understanding of optimal pharmacological approaches for neuropathic pain.

## Data Availability

The original contributions presented in the study are included in the article/Supplementary Material, further inquiries can be directed to the corresponding author.
