# Supplementary figures and images for: Pregabalin vs. gabapentin in the treatment of neuropathic pain: a comprehensive systematic review and meta-analysis of effectiveness and safety
Source: Front Pain Res (Lausanne). 2025 Jan 7;5:1513597. doi: 10.3389/fpain.2024.1513597 (PMC11747324; doi:10.3389/fpain.2024.1513597)

Supplementary Material

**
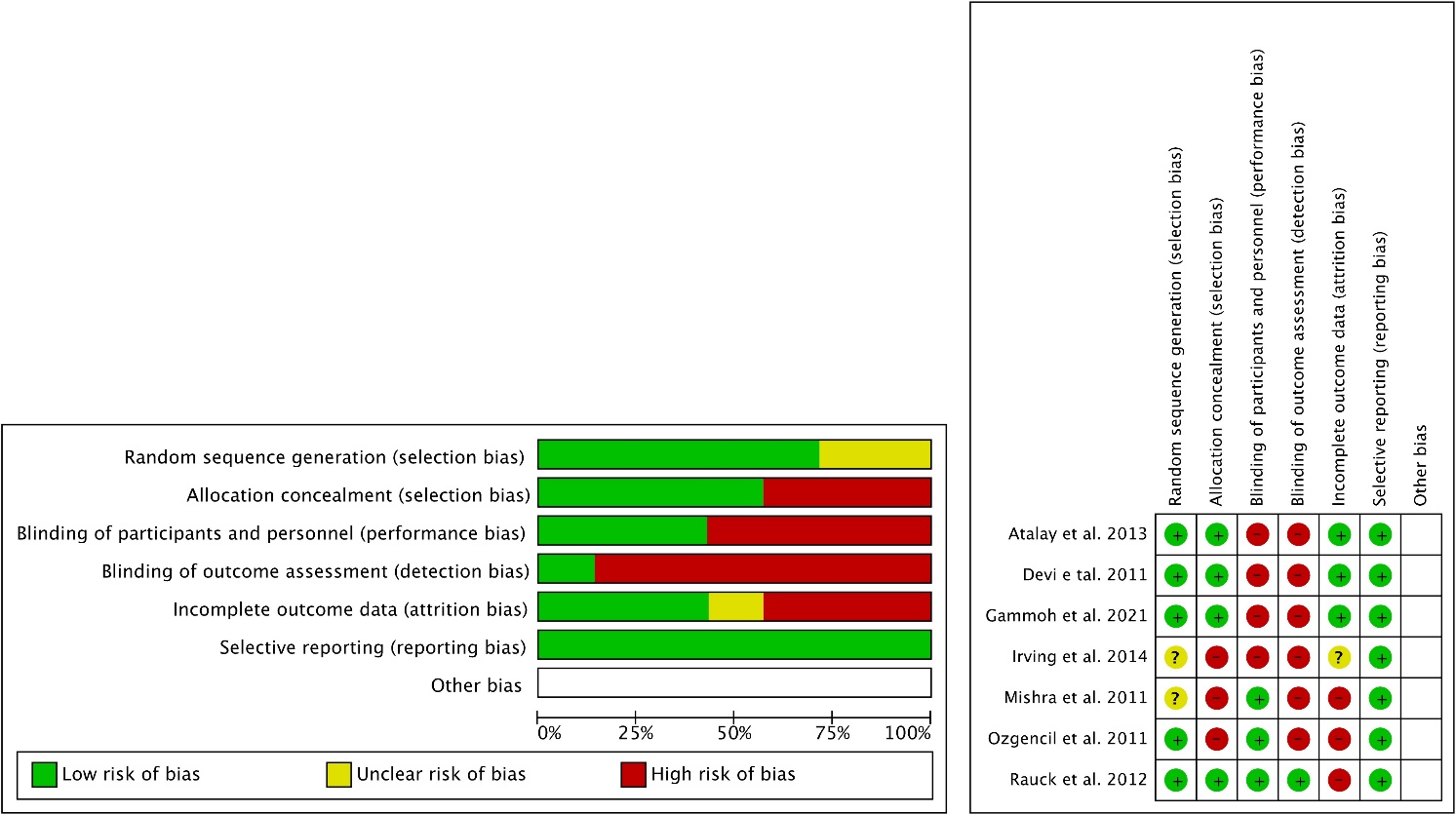
**

**Supplementary figure 1**. Risk of Bias

Supplement: Supplementary file 1 [file Table1.docx]
