# Supplementary material for: Pregabalin vs. gabapentin in the treatment of neuropathic pain: a comprehensive systematic review and meta-analysis of effectiveness and safety
Source: Front Pain Res (Lausanne). 2025 Jan 7;5:1513597. doi: 10.3389/fpain.2024.1513597 (PMC11747324; doi:10.3389/fpain.2024.1513597)
Supplement: Supplementary file 2 [file Table2.docx]

Supplementary Material

**
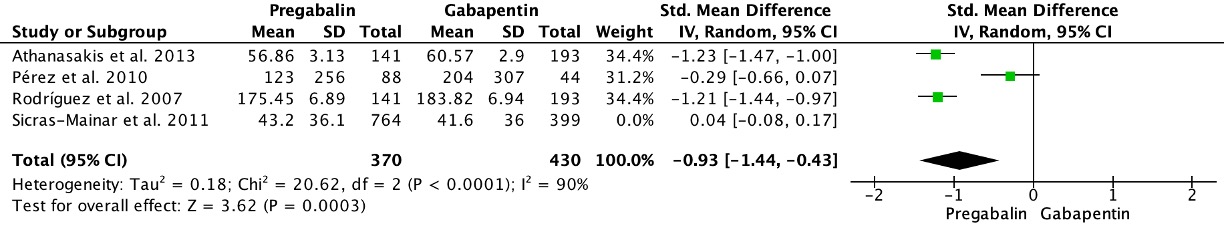
**

**Supplementary figure 2.** The sensitivity analysis, eliminating the study with the highest weight.
