# Supplementary material for: Pregabalin vs. gabapentin in the treatment of neuropathic pain: a comprehensive systematic review and meta-analysis of effectiveness and safety
Source: Front Pain Res (Lausanne). 2025 Jan 7;5:1513597. doi: 10.3389/fpain.2024.1513597 (PMC11747324; doi:10.3389/fpain.2024.1513597)
Supplement: Supplementary file 3 [file Table3.docx]

Supplementary Material

**Supplemnetary File 1.** Pubmed search strategy.

Search: **(pregabalin OR lyrica) AND gabapentin AND neuropathic**

("pregabalin"[MeSH Terms] OR "pregabalin"[All Fields] OR "pregabalin s"[All Fields] OR "pregabaline"[All Fields] OR ("pregabalin"[MeSH Terms] OR "pregabalin"[All Fields] OR "lyrica"[All Fields] OR "pregabalin s"[All Fields] OR "pregabaline"[All Fields])) AND ("gabapentin"[MeSH Terms] OR "gabapentin"[All Fields] OR "gabapentine"[All Fields] OR "gabapentin s"[All Fields]) AND ("neuropathic"[All Fields] OR "neuropathics"[All Fields])

**Translations**

**pregabalin:** "pregabalin"[MeSH Terms] OR "pregabalin"[All Fields] OR "pregabalin's"[All Fields] OR "pregabaline"[All Fields]

**lyrica:** "pregabalin"[MeSH Terms] OR "pregabalin"[All Fields] OR "lyrica"[All Fields] OR "pregabalin's"[All Fields] OR "pregabaline"[All Fields]

**gabapentin:** "gabapentin"[MeSH Terms] OR "gabapentin"[All Fields] OR "gabapentine"[All Fields] OR "gabapentin's"[All Fields]

**neuropathic:** "neuropathic"[All Fields] OR "neuropathics"[All Fields]
