# Supplementary material for: Pregabalin vs. gabapentin in the treatment of neuropathic pain: a comprehensive systematic review and meta-analysis of effectiveness and safety
Source: Front Pain Res (Lausanne). 2025 Jan 7;5:1513597. doi: 10.3389/fpain.2024.1513597 (PMC11747324; doi:10.3389/fpain.2024.1513597)
Supplement: Supplementary file 4 [file Table4.docx]

Supplementary Material

**Supplementary File 2.** Risk of bias judgement.

**Atalay et al. 2013**

1. Patients were randomized into either gabapentin (25 patients) or pregabalin (25 patients) treatment arms using computer-generated random numbers.
2. Figure 1.
3. Not blinded.
4. Not blinded.
5. Figure 1.
6. All kind of outcomes with complete data.

**Devi et al. 2011**

1. Patients who fulfilled the inclusion / exclusion criteria were randomized by computer generated randomization table into the three treatment groups.
2. Figure 1.
3. Not reported.
4. Not reported.
5. Figure 1.
6. All kind of outcomes with complete data.

**Feltner et al. 2003**

1. Eligible patients were randomised by serially numbered envelopes to receive either Pregabalin or Gabapentin for six weeks.
2. Figure 1.
3. Not blinded.
4. Not blinded.
5. Figure 1.
6. All kind of outcomes with complete data.

**Irving et al. 2014**

1. Eligible patients were randomized in a 1 : 1 : 1 ratio to open-label oral treatment with duloxetine, pregabalin or duloxetine plus gabapentin
2. Not reported.
3. Not blinded.
4. Not blinded.
5. Not reported.
6. All kind of outcomes with complete data.

**Mishra et al. 2011**

1. Not reported.
2. Not reported.
3. Double-blind.
4. Not reported.
5. Not reported.
6. All kind of outcomes with complete data.

**Ozgencil et al. 2011**

1. Patients were randomly assigned to three groups using a computer-generated randomisation schedule.
2. Not reported.
3. Double-blind.
4. Not reported.
5. Not reported.
6. All kind of outcomes with complete data.

**Rauck et al. 2012**

1. Table 1.
2. Table 1.
3. Double-blind.
4. Gabapentin enacarbil was provided as 600 mg tablets with identical-in-appearance placebo tablets to ensure blinding of subjects and investigators. To maintain blinding during the maintenance phase, all subjects were instructed to take the study medication as indicated in Table 1 in the morning, afternoon, and evening.
5. Not reported.
6. All kind of outcomes with complete data.
